# P-906. Comparative and Diagnostic Accuracy of Procalcitonin and White Blood Cell Count in Guiding Antibiotic Therapy

**DOI:** 10.1093/ofid/ofaf695.1112

**Published:** 2026-01-11

**Authors:** Roshni Murali, Suhail Hassan Jalal, Suresh Kumar Dorairajan

**Affiliations:** The Tamil Nadu Dr. M.G.R. Medical University, Chennai, Tamil Nadu, India; The Tamil Nadu Dr. M.G.R. Medical University, Chennai, Tamil Nadu, India; The Madras Medical Mission Hospital, Chennai, Tamil Nadu, India

## Abstract

**Background:**

Elevated inflammatory biomarkers such as white blood cell count (WBC) and procalcitonin (PCT) are frequently used to guide empirical antibiotic therapy. However, prescribing antibiotics based solely on biomarker elevation without microbiological confirmation may contribute to inappropriate antibiotic use. This study evaluates the diagnostic performance of WBC and PCT in predicting bacterial infections and examines antibiotic prescribing patterns based on these biomarkers in a tertiary care hospital.

**Fig 1.1:**
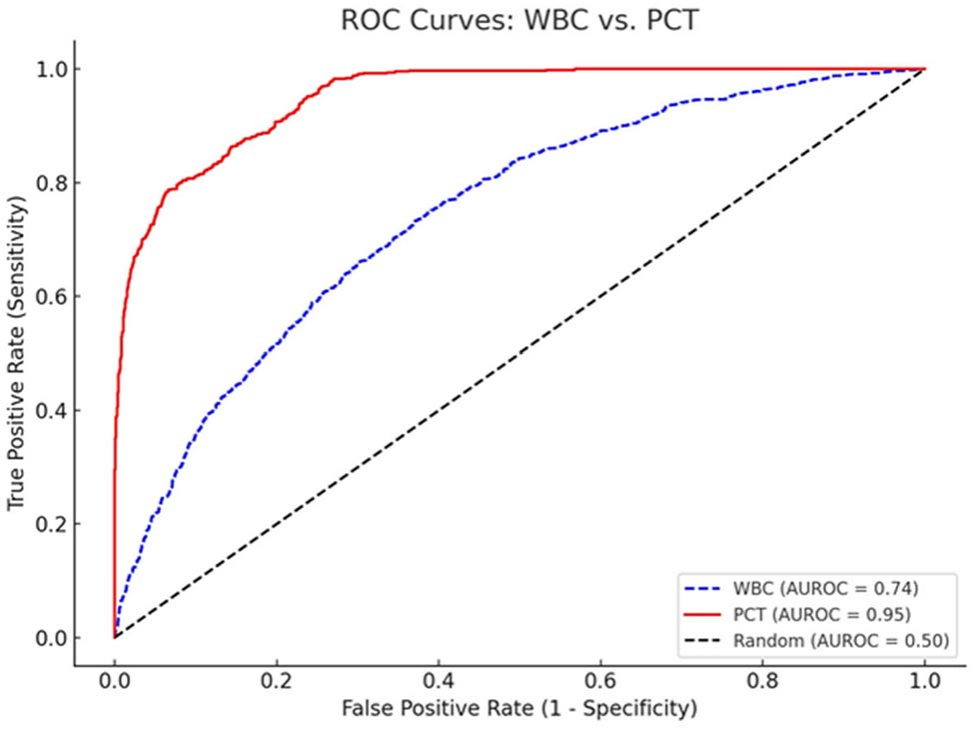
AUROC CURVE

**Figure:**
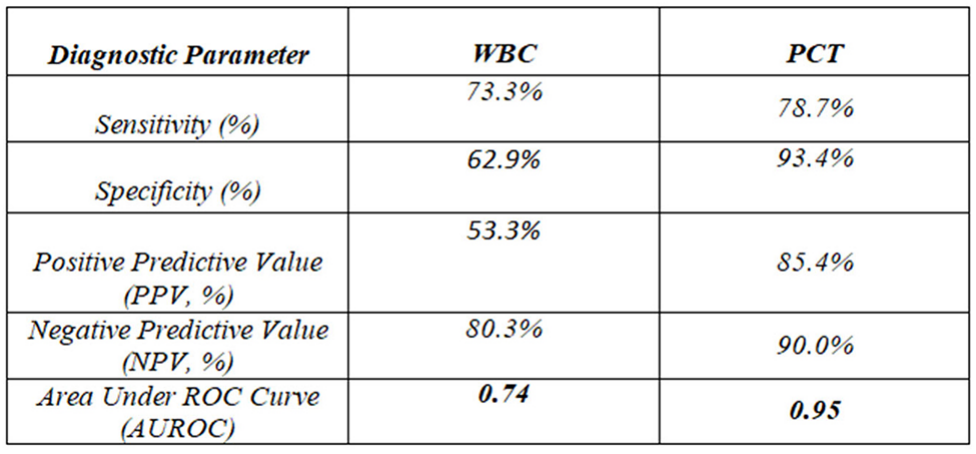

**Methods:**

A prospective observational study was conducted over six months at a tertiary care hospital. Adult patients with elevated WBC ( >12,000/mm³) or PCT ( >0.5 ng/mL) without confirmed infection were included. Data on demographics, biomarker levels, antibiotic prescriptions, and clinical outcomes were collected. Diagnostic performance was assessed by calculating sensitivity, specificity, positive predictive value, negative predictive value, and AUROC. Statistical analysis included descriptive statistics and AUROC analysis

**Results:**

In this study, 3,425 patients with elevated white blood cell count (WBC >12,000/mm³) were assessed, with 1,254 (36.6%) receiving antibiotics. Of 2,086 patients with elevated procalcitonin (PCT >0.5 ng/mL), 685 (32.8%) were prescribed antibiotics. Positive bacterial cultures were found in 397 (31.6%) of the WBC group and 399 (58.2%) of the PCT group. Table 1.1 and Figure 1.1 present the AUROC curve and diagnostic performance metrics, including sensitivity, specificity, PPV, NPV, and AUROC for both biomarkers.

**Conclusion:**

In conclusion, PCT exhibited superior diagnostic performance compared to WBC, showing higher sensitivity and specificity in identifying bacterial infections. These findings suggest that PCT may serve as a more reliable tool for guiding antibiotic therapy, potentially enhancing antimicrobial stewardship and minimizing unnecessary antibiotic use.

**Disclosures:**

All Authors: No reported disclosures

